# Supplementary material for: The Varietal Influence of Flavour Precursors from Grape Marc on Monoterpene and C13-Norisoprenoid Profiles in Wine as Determined by Membrane-Assisted Solvent Extraction (MASE) GC-MS
Source: Molecules. 2022 Mar 22;27(7):2046. doi: 10.3390/molecules27072046 (PMC9000514; doi:10.3390/molecules27072046)
Supplement: Supplementary file 1 [file molecules-27-02046-s001.zip › molecules-1635256-supplementary.pdf]

molecules

MDPI

Supplementary materials for

The varietal influence of flavour precursors from grape marc on monoterpene and C_{13}-norisoprenoid profiles in wine as determined by membrane-assisted solvent extraction (MASE) GC-MS

Lisa Pisaniello, Flynn Watson, Tracey Siebert, Leigh Francis and Josh Hixson

molecules

MDPI

Table S1: Red wine calibration and validation data for membrane-assisted solvent extraction (MASE) GC-MS method, as determined in duplicate in commercially available Shiraz (2018, 13.5% alcohol) or Pinot Noir (2017, 13.0% alcohol).

Analyte	Retention time (mins)	Internal standard	Ions monitored (m/z)		Linearity		Limits (µg/L)		Low Spike (n = 7)		High Spike (n = 7)	
			Quantifier	Qualifiers (%)	R^2	Range (µg/L)	Detection	Quantitation	Recovery (%)	Repeatability (RSD %)	Recovery (%)	Repeatability (RSD %)
Limonene	13.86	d_6-1,8-Cineole	136	93 (230), 68 (255)	0.993	0, 0.5 - 100 µg/L	0.03	0.11	93.0	4.8	89.8	4.2
1,8-Cineole	14.41		111	154 (13), 126 (128)	0.9997	0, 0.5 - 100 µg/L	0.03	0.1	100.1	2.5	100.0	0.7
Terpinoline	15.55	d_6-Linalool	136	121 (106), 105 (28)	0.99	0, 0.5 - 100 µg/L	0.01	0.03	91.0	7.3	90.4	7.2
Linalool	15.73		71	136 (40), 121 (19)	0.9989	0, 0.5 - 100 µg/L	0.04	0.14	97.8	6.0	100.6	1.6
cis-Rose oxide	16.18	d_6-α-Terpineol	139	154 (14), 140 (10)	0.9946	0, 0.5 - 100 µg/L	0.01	0.02	107.7	7.8	104.0	6.5
trans-Rose oxide	16.8		139	154 (10), 140 (11)	0.9942	0, 0.5 - 100 µg/L	0.01	0.03	106.9	8.3	107.7	6.5
α-Terpineol	19.41		136	93 (110), 59 (114)	0.9999	0, 0.5 - 100 µg/L	0.1	0.35	104.8	8.5	101.8	1.3
β-Citronellol	19.49	d_2-β-Citronellol	123	156 (38), 69 (440)	0.9939	0, 0.5 - 100 µg/L	0.11	0.37	104.0	4.8	98.1	2.6
Nerol	19.85		139	154 (48), 121 (4200)	0.995	0, 10 - 100 µg/L	0.87	2.89	96.0	9.5	106.6	2.3
Geraniol	20.62	d_7-Geraniol	136	93 (440), 123 (400)	0.9845	0, 10 - 100 µg/L	1.49	4.98	99.6	4.8	100.6	3.0
β-Damascenone	25.43	d_4-β-Damascenone	69	190 (36), 175 (16)	0.9992	0, 0.1 - 100 µg/L	0	0.01	96.5	1.6	103.1	2.5
α-Ionone	26.21	d_3-α-Ionone	136	121 (125), 93 (210)	0.9993	0, 0.1 - 100 µg/L	0.15	0.5	104.7	4.4	101.7	1.4
β-Ionone	28.15	d_3-β-Ionone	177.1	192 (13), 43 (6)	0.9982	0, 0.1 - 100 µg/L	0.01	0.02	85.1	3.0	101.4	1.0

Table S2: Geraniol glucoside, monoterpene pentosyl-glucoside (MT PGs) and monoterpene rhamnosyl-glucoside (MT RGs) concentrations in Chardonnay wine with and without a 0.4 g/L addition of Muscat Gordo marc extract, analysed periodically over 154 days of storage at 15 °C.

Sample	Replicate	Days of storage 41			64			99			126			154		
		Geraniol glucoside	MT PGs*	MT RGs*	Geraniol glucoside	MT PGs*	MT RGs*	Geraniol glucoside	MT PGs*	MT RGs*	Geraniol glucoside	MT PGs*	MT RGs*	Geraniol glucoside	MT PGs*	MT RGs*
Chardonnay Control	1	18.07	4.75	< LOD^	16.80	4.80	< LOD	21.36	4.31	< LOD	18.86	4.59	< LOD	19.64	5.10	< LOD
	2	16.31	4.90	< LOD	17.48	5.33	< LOD	21.89	4.77	< LOD	19.50	4.57	< LOD	20.01	5.11	< LOD
	3	18.38	4.85	< LOD	17.46	5.30	< LOD	19.67	4.59	< LOD	18.67	4.67	< LOD	21.34	5.01	< LOD
Chardonnay Spiked	1	517.15	143.24	24.18	583.24	138.20	23.87	628.97	125.40	22.54	722.64	115.73	23.01	736.87	108.89	21.17
	2	526.63	142.06	23.93	605.33	133.93	23.15	626.11	124.37	23.53	676.16	114.74	21.55	742.54	103.81	21.23
	3	503.53	142.01	23.36	596.38	139.75	24.27	664.97	129.25	24.29	719.01	115.14	22.34	745.18	111.01	22.84

* Determined using deuterated syringol gentiobioside as an internal standard, quoted in syringol gentiobioside equivalents.

^ Limit of detection (LOD) for monoterpene rhamnose-glucoses (MT RGs) was 0.29 µg/L. Replacement value for samples '< LOD' was 0.14 µg/L.

Table S3: Concentration of selected monoterpenes and C_{13}-norisoprenoids in Chardonnay wine with and without a 0.4 g/L addition of Muscat Gordo marc extract, analysed periodically over 154 days of storage at 15 °C.

		41 Days of storage Concentration (μg/L)															
**Sample**	**Replicate**	**Limonene**	**1,8-Cineole**	**Terpinolene**	**Linalool**	**cis-Rose Oxide**	**trans-Rose Oxide**	**α-Terpineol**	**β-Citronellol**	**Nerol**	**Geraniol**	**cis-Vitispirane**	**TDN**	**β-Damascenone**	**α-Ionone**	**β-Ionone**	**Wine Lactone**
Chardonnay Control	1	< LOQ	< LOQ	< LOD	< LOD	< LOD	< LOD	5.017	< LOD	< LOD	< LOQ	0.263	0.624	< LOQ	< LOD	0.106	< LOD
	2	< LOQ	< LOQ	< LOD	< LOQ	< LOD	< LOD	5.234	< LOQ	< LOD	< LOQ	0.289	0.371	< LOQ	< LOD	0.086	< LOD
	3	< LOQ	< LOQ	< LOD	< LOQ	< LOD	< LOD	5.119	< LOQ	< LOD	< LOQ	0.394	0.422	< LOQ	< LOD	0.068	< LOD
Chardonnay Spiked	1*	-	-	-	-	-	-	-	-	-	-	-	-	-	-	-	-
	2	0.806	< LOQ	< LOQ	257.951	0.229	0.075	105.6	< LOD	11.26	50.76	0.631	0.540	2.618	< LOD	0.103	< LOD
	3	0.761	< LOQ	< LOQ	263.997	0.245	0.082	108.1	< LOQ	9.066	40.75	0.552	0.455	2.672	< LOQ	0.106	< LOD
Limit of Quantitation (LOQ, μg/L)^		0.113	0.286	0.933	11.320				8.227		5.862			1.104	0.111		
Limit of Detection (LOD, μg/L)^		0.034	0.086	0.280	3.395	0.025	0.021		2.468	1.623	1.758			0.331	0.033		2.033
< LOQ replacement value†		0.073	0.186	0.607	7.358				5.348		3.810			0.718	0.072		
< LOD replacement value‡		0.017	0.043	0.140	1.698	0.013	0.011		1.234	0.812	0.879			0.166	0.017		1.017

* Analysis of Spiked Chardonnay Replicate 1 failed for 'Day 41' samples and provided no data.

		64 Days of storage Concentration (μg/L)															
**Sample**	**Replicate**	**Limonene**	**1,8-Cineole**	**Terpinolene**	**Linalool**	**cis-Rose Oxide**	**trans-Rose Oxide**	**α-Terpineol**	**β-Citronellol**	**Nerol**	**Geraniol**	**cis-Vitispirane**	**TDN**	**β-Damascenone**	**α-Ionone**	**β-Ionone**	**Wine Lactone**
Chardonnay Control	1	0.032	0.060	0.175	7.352	0.020	< LOD	5.142	2.346	< LOD	< LOQ	0.402	0.052	1.243	< LOD	0.042	< LOQ
	2	0.031	0.066	0.176	7.569	< LOQ	< LOD	5.094	2.177	< LOD	< LOQ	0.374	0.063	1.237	< LOD	0.045	< LOQ
	3	0.026	0.061	0.210	7.951	0.020	< LOD	5.221	1.803	< LOD	< LOQ	0.460	0.094	1.235	< LOQ	0.040	< LOQ
Chardonnay Spiked	1	1.095	0.078	0.952	394.274	0.266	0.056	163.481	< LOQ	14.932	63.365	1.150	0.220	3.764	< LOQ	0.061	< LOQ
	2	1.161	0.073	1.127	461.388	0.201	0.042	157.601	< LOQ	11.431	72.651	0.776	0.167	3.811	0.065	0.096	0.307
	3	1.075	0.080	0.912	365.031	0.247	0.054	155.121	< LOQ	11.257	54.528	1.121	0.220	3.423	< LOQ	0.061	< LOQ
Limit of Quantitation (LOQ, μg/L)^						0.018			1.795		1.262				0.047		0.207
Limit of Detection (LOD, μg/L)^						0.005	0.004		0.539	0.693	0.379				0.014		0.062
< LOQ replacement value†						0.012			1.167		0.821				0.031		0.135
< LOD replacement value‡						0.003	0.002		0.270	0.347	0.190				0.007		0.031

		99 Days of storage Concentration (μg/L)															
**Sample**	**Replicate**	**Limonene**	**1,8-Cineole**	**Terpinolene**	**Linalool**	**cis-Rose Oxide**	**trans-Rose Oxide**	**α-Terpineol**	**β-Citronellol**	**Nerol**	**Geraniol**	**cis-Vitispirane**	**TDN**	**β-Damascenone**	**α-Ionone**	**β-Ionone**	**Wine Lactone**
Chardonnay Control	1	< LOQ	0.152	< LOD	4.496	0.020	< LOD	5.646	2.803	< LOQ	< LOQ	0.355	0.039	1.046	0.351	0.015	< LOD
	2	< LOQ	0.143	< LOD	4.659	0.021	< LOD	5.064	2.510	< LOQ	< LOQ	0.312	0.039	1.064	0.346	0.014	< LOD
	3	< LOQ	0.147	< LOD	4.211	0.022	< LOD	4.310	2.516	< LOQ	< LOQ	0.336	0.058	0.955	0.369	0.023	< LOQ
Chardonnay Spiked	1	1.014	0.129	0.450	503.915	0.261	0.038	243.648	2.713	22.501	44.872	1.309	0.182	3.396	0.355	0.032	< LOD
	2	1.117	0.185	0.568	606.665	0.262	0.038	265.290	< LOQ	29.577	68.111	1.141	0.138	3.726	0.370	0.030	< LOD
	3	1.192	0.143	0.620	555.144	0.294	0.043	242.506	< LOQ	26.930	45.254	1.120	0.136	3.344	0.389	0.032	< LOD
Limit of Quantitation (LOQ, μg/L)^		0.118							2.237	1.202	2.254						0.173
Limit of Detection (LOD, μg/L)^		0.035		0.028			0.005		0.671	0.361	0.676						0.052
< LOQ replacement value†		0.076							1.454	0.782	1.465						0.113
< LOD replacement value‡		0.018		0.014			0.003		0.336	0.181	0.338						0.026

^ LOD and/or LOQ only specified where samples are quoted below that limit, or for calculation of replacement values.

† Replacement value determined as mean of LOQ and LOD.

‡ Replacement value determined as mean of LOD and zero.

molecules

MDPI

Table S3 continued: Concentration of selected monoterpenes and C_{13}-norisoprenoids in Chardonnay wine with and without a 0.4 g/L addition of Gordo marc extract, analysed periodically over 154 days of storage at 15 °C.

		126 Days of storage Concentration (µg/L)															
**Sample**	**Replicate**	**Limonene**	**1,8-Cineole**	**Terpinolene**	**Linalool**	**cis-Rose Oxide**	**trans-Rose Oxide**	**α-Terpineol**	**β-Citronellol**	**Nerol**	**Geraniol**	**cis-Vitispirane**	**TDN**	**β-Damascenone**	**α-Ionone**	**β-Ionone**	**Wine Lactone**
Chardonnay Control	1	< LOQ	0.096	< LOD	5.217	0.015	< LOD	5.637	4.473	0.741	1.684	0.520	< LOD	1.125	< LOQ	0.030	< LOD
	2	0.056	0.136	< LOD	5.128	0.018	< LOD	5.838	3.964	0.751	1.600	0.581	< LOD	1.059	< LOQ	0.027	< LOD
	3	0.053	0.112	< LOD	5.278	0.015	< LOD	5.597	4.207	0.606	1.971	0.503	< LOD	1.133	< LOQ	0.030	< LOD
Chardonnay Spiked	1	1.442	0.136	0.897	588.100	0.226	0.041	279.300	4.280	54.475	92.070	2.005	0.155	3.655	< LOQ	0.055	< LOD
	2	1.488	1.488	0.696	560.400	0.234	0.037	279.750	4.015	45.850	87.385	1.969	< LOQ	3.674	< LOQ	0.049	< LOD
	3	1.333	1.333	0.688	541.550	0.218	0.039	278.700	3.928	42.500	84.425	2.099	0.161	3.612	< LOQ	0.042	< LOD
Limit of Quantitation (LOQ, µg/L)^		0.053											0.135		0.042		
Limit of Detection (LOD, µg/L)^		0.016		0.027			0.003						0.041		0.012		0.097
< LOQ replacement value†		0.035											0.088		0.027		
< LOD replacement value‡		0.008		0.014			0.002						0.021		0.006		0.049

		154 Days of storage Concentration (µg/L)															
**Sample**	**Replicate**	**Limonene**	**1,8-Cineole**	**Terpinolene**	**Linalool**	**cis-Rose Oxide**	**trans-Rose Oxide**	**α-Terpineol**	**β-Citronellol**	**Nerol**	**Geraniol**	**cis-Vitispirane**	**TDN**	**β-Damascenone**	**α-Ionone**	**β-Ionone**	**Wine Lactone**
Chardonnay Control	1	< LOQ	0.172	< LOD	6.387	0.047	< LOQ	6.854	3.485	0.879	2.768	0.408	< LOD	1.261	< LOQ	0.041	< LOD
	2	< LOQ	0.167	< LOQ	6.399	0.050	< LOQ	6.650	2.732	1.080	2.435	0.422	< LOD	1.234	< LOD	0.047	< LOD
	3	< LOQ	0.163	< LOQ	6.146	0.043	< LOQ	6.547	3.098	0.770	2.133	0.460	< LOD	1.319	< LOD	0.044	< LOD
Chardonnay Spiked	1	1.096	0.175	1.112	724.550	0.301	0.051	375.000	2.783	72.340	124.900	1.786	< LOD	4.008	< LOQ	0.062	< LOD
	2	1.054	0.195	1.172	750.250	0.297	0.055	369.950	2.396	80.745	138.850	1.772	< LOD	4.058	< LOQ	0.055	< LOD
	3	1.011	0.910	1.048	709.350	0.323	0.058	373.650	2.723	74.155	123.850	1.859	< LOD	4.214	< LOQ	0.060	< LOD
Limit of Quantitation (LOQ, µg/L)^		0.144		0.135			0.013								0.073		
Limit of Detection (LOD, µg/L)^		0.043		0.040			0.004						0.041		0.022		0.336
< LOQ replacement value†		0.094		0.088			0.009								0.048		
< LOD replacement value‡		0.022		0.020			0.002						0.021		0.011		0.168

^ LOD and/or LOQ only specified where samples are quoted below that limit, or for calculation of replacement values.

† Replacement value determined as mean of LOQ and LOD.

‡ Replacement value determined as mean of LOD and zero.

Table S4: Grape samples collected and used to create marc extracts. Analysis of geraniol glucoside, monoterpene pentosyl-glucosides (MT PGs) and monoterpene rhamnosyl-glucosides (MT RGs) conducted via LC-MS/MS directly on juice or by dissolving approximately 20 mg of extract in 5 mL of water.

Variety	Type	Processing masses (kg)			Juice volume (L)^	Mass of extract [illegible]	Extract yield		Extract analysis (∝g/g of extract)			Juice analysis (∝g/L)		
		Grape weight	Marc produced	Juice produced			Per marc weight (g/kg)	Per grape weight [illegible]	Geraniol glucoside	MT PGs*	MT RGs*	Geraniol glucoside	MT PGs*	MT RGs*
Muscat a PGB	Floral	10.209	4.369	5.840	5.458	1.21	0.277	0.119	1200.99	121.08	74.66	122.52	335.69	47.56
Viognier	Floral	9.805	5.112	4.693	4.386	1.30	0.254	0.133	216.66	33.71	< LOQ	20.90	61.54	8.76
Riesling	Floral	10.046	4.948	5.099	4.765	0.81	0.164	0.081	185.66	33.26	9.78	< LOD	33.57	5.04
Muscat Gordo	Floral	10.223	5.597	4.626	4.324	1.45	0.259	0.142	2082.21	223.49	56.89	176.01	359.00	87.19
Gerwurztraminer	Floral	10.246	4.890	5.356	5.005	1.61	0.329	0.157	1305.81	52.48	24.36	998.13	118.35	14.68
Chardonnay	Non-floral	9.581	4.298	5.283	4.937	1.48	0.344	0.154	< LOD[#,‡]	< LOQ[†,‡]	< LOQ	< LOD	10.89	< LOD
Semillon	Non-floral	10.256	4.439	5.817	5.437	1.42	0.320	0.138	44.60	5.46	< LOQ	< LOQ	18.05	< LOQ
Verdelho	Non-floral	10.089	4.449	5.640	5.271	2.03	0.456	0.201	27.43	< LOQ	< LOQ	6.36	55.95	< LOQ
Sauvignon Blanc	Non-floral	10.249	4.222	6.027	5.633	1.56	0.369	0.152	85.00	8.04	< LOQ	< LOQ	33.34	5.90
Shiraz	Non-floral	9.966	4.244	5.722	5.348	2.14	0.504	0.215	93.33	6.83	< LOQ	82.09	52.45	5.59
Cabernet Sauvignon	Non-floral	9.156	4.535	4.621	4.319	1.70	0.375	0.186	< LOQ	< LOQ	< LOQ	< LOD	5.38	< LOQ

^ Juice volume calculated from weight using an average juice density of 1.07 kg/L.

* Determined using deuterated syringol gentiobioside as an internal standard, quoted in syringol gentiobioside equivalents.

[#] Analytical limits for geraniol glucoside: LOQ 5.0 ∝g/L; LOD, 1.00 ∝g/L determined in solution of extracts or juice. Equates to roughly 25 and 5 ∝g/g of extract, respectively.

[†] Analytical limits for disaccharides: LOQ 1.0 ∝g/L and LOD 0.3 ∝g/L in dissoved extracts (Equates to roughly 5 and 1 ∝g/g of extract); LOQ 5.0 ∝g/L and LOD 1.7 ∝g/L in juice.

[‡] Replacement value for < LOQ samples was the mean of the LOQ and LOD. For < LOD samples, the mean of the LOD and zero, in the matrix analysed in (juice or dissolved extract).

molecules

MDPI

Table S5: Analysis of selected monoterpenes and C_{13}-norisoprenoids by GC-MS after hydrolysis of marc extracts at pH 1 and 100 °C for one hour. Marc extracts were dissolved in water at approximately 400 mg/L (20 mg in 5 mL water) and hydrolysed in singlicate.

Variety	Type	Concentration after hydrolysis (∝g/g of extract)				
		**Linalool**	**a-Terpineol**	**TDN**	**b-Damascenone**	**b-Ionone**
Muscat a PGB	Floral	437.6	766.5	63.1	53.1	10.7
Viognier	Floral	90.5	118.3	52.0	39.4	11.4
Riesling	Floral	213.8	256.6	421.2	112.3	10.5
Muscat Gordo	Floral	1909.0	1677.2	203.8	132.4	11.3
Gerwurztraminer	Floral	161.2	153.3	68.7	66.4	< LOD*
Chardonnay	Non-floral	13.1	20.1	75.5	102.5	11.5
Semillon	Non-floral	62.8	43.3	120.2	79.4	< LOD
Verdelho	Non-floral	69.5	48.9	188.5	52.7	11.5
Sauvignon Blanc	Non-floral	111.9	78.8	119.2	119.8	10.5
Shiraz	Non-floral	34.5	30.8	130.5	72.1	12.0
Cabernet Sauvignon	Non-floral	35.0	26.7	92.5	106.7	< LOD

* Limit of Detection for b-ionone (5 ∝g/L) was determined in solution of hydrolysate and equates to roughly 2.6 ∝g/g of extract. Replacement value for '< LOD' samples was 2.5 ∝g/L in the analysis matrix.

Table S6: Concentration of marc extracts spiked into commercial Chardonnay wine, and calculated spike concentration of bound monoterpenes in the wines prior to storage.

Sample	Type	Extract spike (g/L)	Spiked concentration (∝g/L)		
			Geraniol glucoside	MT PGs*	MT RGs*
2016 Gewurztraminer ^	Floral	0.401	1002.50	-	-
Muscat a PGB	Floral	0.396	475.68	47.95	29.57
Viognier	Floral	0.405	87.74	13.65	1.21[‡]
Riesling	Floral	0.402	74.71	13.38	3.94
Muscat Gordo	Floral	0.402	836.75	89.81	22.86
Gerwurztraminer	Floral	0.396	517.56	20.80	9.66
Chardonnay	Non-floral	0.400	0.80[†]	0.20[‡]	1.20[‡]
Semillon	Non-floral	0.404	18.02	2.21	1.21[‡]
Verdelho	Non-floral	0.402	11.02	0.80[‡]	0.80[‡]
Sauvignon Blanc	Non-floral	0.399	33.95	3.21	1.20[‡]
Shiraz	Non-floral	0.403	37.57	2.75	1.21[‡]
Cabernet Sauvignon	Non-floral	0.400	5.20[‡]	1.20[‡]	1.20[‡]

* Determined using deuterated syringol gentiobioside as an internal standard, quoted in syringol gentiobioside equivalents.

^ Monoterpene disaccharides not determined in original analysis of this extract.

[†] Calculated from a value replacing a '< LOD' measurement.

‡ Calculated from a value replacing a '< LOQ' measurement.

molecules

MDPI

Table S7: Geraniol glucoside, monoterpene glucoside (MT Glu), monoterpene pentosyl-glucoside (MT PGs) and monoterpene rhamnosyl-glucoside (MT RGs) concentrations in control Chardonnay wine and Chardonnay wine with 0.4 g/L additions of marc extract from different varieties, analysed after 6-months of storage at 15 °C.

Sample	Type	Replicate	Concentration (∝g/L)			
			Geraniol glucoside	MT Glu^	MT PGs*	MT RGs*
Control Wine	Control	1	22.37	26.40	6.61	< LOD[#]
		2	21.26	26.12	6.39	< LOD
		3	21.66	27.06	4.35	< LOD
2016 Gewurztraminer	Floral	1	1940.73	2158.38	154.11	53.13
		2	2071.00	2281.75	159.87	54.22
		3	2125.55	2355.50	162.07	53.70
Muscat Gordo	Floral	1	1513.99	1645.66	262.16	59.55
		2	1609.18	1752.16	259.44	58.82
		3	1626.13	1751.52	272.90	61.07
Muscat a PGB	Floral	1	968.99	1343.45	130.27	84.65
		2	1000.80	1342.32	136.91	88.30
		3	953.67	1318.94	128.58	82.14
Riesling	Floral	1	163.06	182.97	41.80	8.62
		2	133.18	148.46	32.42	7.00
		3	144.26	160.26	34.77	7.80
Gewurztraminer	Floral	1	731.66	818.37	67.09	17.80
		2	795.46	892.67	64.65	17.41
		3	764.12	860.21	63.90	16.37
Viognier	Floral	1	222.86	237.83	32.78	3.56
		2	152.46	165.89	23.87	2.09
		3	161.53	172.63	24.67	2.56
Cabernet Sauvignon	Non-floral	1	32.78	39.18	7.48	< LOQ
		2	29.33	34.76	6.00	< LOQ
		3	28.78	34.02	6.54	< LOQ
Chardonnay	Non-floral	1	36.93	44.05	11.77	< LOD
		2	37.05	44.51	12.62	< LOQ
		3	37.61	45.20	12.84	< LOQ
Sauvignon Blanc	Non-floral	1	98.99	107.80	13.05	2.63
		2	98.95	106.73	13.23	2.72
		3	103.64	112.90	13.36	2.51
Semillon	Non-floral	1	83.69	93.96	15.34	2.07
		2	81.26	88.02	15.65	1.95
		3	85.85	95.10	15.94	1.61
Shiraz	Non-floral	1	95.78	105.77	18.53	< LOQ
		2	95.44	105.83	18.11	1.45
		3	94.18	104.68	18.58	1.75
Verdelho	Non-floral	1	61.75	69.01	13.45	< LOQ
		2	55.24	63.96	12.63	< LOQ
		3	52.09	59.78	11.64	< LOQ

^ Determined using deuterated geraniol glucoside as an internal standard, quoted in geraniol glucoside equivalents.

* Determined using deuterated syringol gentiobioside as an internal standard, quoted in syringol gentiobioside equivalents.

[#] Analytical limits for MT RGs was: LOQ, 1.38 ∝g/L; LOD, 0.41 ∝g/L. Replacement values: < LOQ, 0.90 ∝g/L; < LOD, 0.21 ∝g/L.

molecules

MDPI

Table S8: Concentration of selected monoterpenes and C_{13}-norisoprenoids in control Chardonnay wine and Chardonnay wine with 0.4 g/L additions of marc extract from different varieties, analysed after 6-months of storage at 15 °C.

Sample	Category	Replicate	Concentration (∝g/L)															
			Limonene	1,8-Cineole	Terpinolene	Linalool	cis-Rose Oxide	trans-Rose Oxide	a-Terpineol	b-Citronellol	Nerol	Geraniol	cis-Vitispirane	TDN	b-Damascenone	a-Ionone	b-Ionone	Wine Lactone
Control Wine	Control	1	< LOQ	< LOQ	< LOD	5.777	0.016	< LOD	5.702	4.170	< LOQ	2.161	0.112	< LOD	1.398	< LOD	0.032	< LOD
		2	0.168	< LOQ	< LOD	6.067	< LOQ	< LOD	5.640	4.163	< LOQ	2.001	0.123	< LOD	1.293	< LOD	0.030	< LOD
		3*	-	-	-	-	-	-	-	-	-	-	-	-	-	-	-	-
2016 Gewurztraminer	Floral	1	< LOQ	< LOQ	< LOD	41.419	0.170	0.025	31.891	2.895	5.249	12.854	1.100	< LOD	7.520	< LOQ	0.025	< LOD
		2	0.184	< LOQ	< LOD	39.382	0.146	< LOQ	30.296	3.286	5.575	14.167	1.176	< LOD	7.068	< LOQ	0.029	< LOD
		3	0.197	< LOQ	< LOD	41.612	0.120	< LOQ	29.777	2.817	4.192	12.613	1.480	< LOD	7.429	< LOQ	0.039	< LOD
Muscat Gordo	Floral	1	2.947	0.396	2.558	1732.692	0.586	0.092	977.289	12.015	158.374	840.459	2.087	< LOD	16.128	< LOQ	0.187	< LOQ
		2	3.076	< LOQ	3.172	1762.440	0.551	0.082	902.861	12.380	139.209	719.649	2.460	< LOQ	15.643	< LOQ	0.193	< LOQ
		3	3.603	0.390	3.709	1736.587	0.558	0.082	929.243	11.311	135.572	720.433	2.148	< LOQ	14.739	< LOQ	0.185	< LOD
Muscat a PGB	Floral	1	1.198	< LOQ	1.458	543.755	0.802	0.131	337.813	3.263	42.966	94.221	1.102	< LOQ	7.702	< LOQ	0.046	< LOD
		2	1.148	< LOQ	1.253	567.739	0.756	0.126	350.511	3.859	44.499	101.725	1.008	< LOD	7.438	< LOQ	0.037	< LOD
		3	0.771	< LOQ	0.793	570.537	0.705	0.113	321.542	4.030	41.704	101.361	0.935	< LOD	7.049	< LOD	< LOQ	< LOD
Riesling	Floral	1	0.387	< LOQ	< LOQ	196.268	0.054	< LOQ	120.595	2.770	14.158	33.353	3.641	< LOQ	19.581	< LOQ	0.154	< LOQ
		2	0.275	< LOQ	< LOQ	158.956	0.030	< LOD	100.839	2.779	7.474	20.630	3.260	< LOD	15.340	< LOQ	0.126	< LOD
		3*	-	-	-	-	-	-	-	-	-	-	-	-	-	-	-	-
Gewurztraminer	Floral	1	0.214	< LOQ	< LOD	17.255	0.538	0.074	16.465	2.862	1.942	5.525	0.739	< LOD	7.267	< LOQ	0.051	< LOD
		2	< LOQ	< LOQ	< LOD	16.885	0.484	0.069	16.091	3.138	1.828	5.279	0.792	< LOD	7.168	< LOQ	0.048	< LOD
		3	< LOQ	< LOQ	< LOD	17.088	0.577	0.085	16.525	4.135	2.078	5.512	0.794	< LOD	6.390	< LOQ	0.029	< LOQ
Viognier	Floral	1	0.363	< LOQ	< LOQ	205.302	0.137	0.026	123.429	4.598	13.436	34.727	0.902	< LOD	8.795	< LOQ	0.123	< LOQ
		2	0.393	< LOQ	< LOQ	134.314	0.081	< LOQ	79.168	2.765	8.976	26.266	0.727	< LOD	6.097	< LOQ	0.085	< LOQ
		3	0.368	< LOQ	< LOQ	135.268	0.108	< LOQ	82.126	4.309	9.548	23.623	0.789	< LOD	5.399	< LOQ	0.095	< LOD
Cabernet Sauvignon	Non-floral	1	< LOQ	< LOQ	< LOQ	6.141	0.022	< LOD	8.037	2.665	< LOQ	2.516	0.925	< LOD	13.667	< LOQ	< LOQ	< LOD
		2	0.211	< LOQ	< LOD	5.730	0.021	< LOD	8.074	2.629	< LOQ	2.407	0.839	< LOD	10.646	< LOD	0.032	< LOD
		3	< LOQ	< LOQ	< LOD	6.155	0.023	< LOD	8.284	2.737	< LOQ	2.219	0.916	< LOD	9.147	< LOQ	0.043	< LOD
Chardonnay	Non-floral	1	< LOQ	< LOQ	< LOD	11.765	0.023	< LOD	11.537	2.385	< LOQ	3.211	0.743	< LOD	9.818	< LOQ	0.058	< LOD
		2	< LOQ	< LOQ	< LOD	11.538	0.026	< LOD	11.205	< LOQ	< LOQ	3.219	0.734	< LOD	9.696	< LOQ	0.066	< LOD
		3	0.198	< LOQ	< LOQ	12.343	0.027	< LOD	11.560	2.526	< LOQ	3.502	0.730	< LOD	9.047	< LOQ	0.063	< LOD
Sauvignon Blanc	Non-floral	1	< LOQ	< LOQ	< LOD	9.496	0.041	< LOQ	10.901	2.194	< LOQ	3.167	1.218	< LOD	12.320	< LOQ	0.081	< LOD
		2	< LOQ	< LOQ	< LOD	8.884	0.042	< LOD	11.215	2.967	< LOQ	3.071	1.262	< LOD	13.429	< LOQ	0.070	< LOD
		3	< LOQ	< LOQ	< LOD	9.224	0.049	< LOQ	10.772	2.658	< LOQ	3.036	1.275	< LOD	12.824	< LOQ	0.046	< LOD
Semillon	Non-floral	1	< LOQ	< LOQ	< LOQ	9.331	0.036	< LOD	11.004	2.913	< LOQ	3.043	1.245	< LOD	9.195	< LOQ	0.032	< LOD
		2	< LOQ	< LOQ	< LOD	8.603	0.023	< LOD	10.099	2.356	< LOQ	2.981	1.058	< LOD	8.014	< LOQ	0.029	< LOD
		3	< LOQ	< LOQ	< LOD	9.035	0.028	< LOD	9.994	2.788	< LOQ	2.670	1.426	< LOD	7.472	< LOQ	0.055	< LOD
Shiraz	Non-floral	1	< LOQ	< LOQ	< LOD	7.857	0.035	< LOD	9.081	< LOQ	< LOQ	2.581	1.010	< LOD	7.076	< LOQ	0.030	< LOD
		2	< LOQ	< LOQ	< LOD	7.880	0.030	< LOQ	8.788	< LOQ	< LOQ	2.067	1.025	< LOD	6.857	< LOQ	0.038	< LOD
		3	< LOQ	< LOQ	< LOD	8.121	0.031	< LOD	9.039	2.748	1.711	< LOQ	1.012	< LOD	6.293	< LOQ	0.032	< LOD
Verdelho	Non-floral	1	0.222	< LOQ	< LOQ	26.033	0.031	< LOD	20.976	< LOQ	2.201	4.918	0.964	< LOD	3.205	< LOQ	0.050	< LOD
		2	0.197	< LOQ	< LOQ	27.492	0.028	< LOD	21.300	2.504	2.800	5.158	0.989	< LOD	3.179	< LOQ	0.052	< LOD
		3	0.216	< LOQ	< LOQ	30.211	0.030	< LOD	22.870	< LOQ	2.834	3.863	0.991	< LOD	3.716	< LOD	0.052	< LOD
Limit of Quantitation (LOQ, ∝g/L)			0.167	0.299	0.389	0.803	0.014	0.020	0.561	2.031	1.569	1.881	0.043	1.065	0.072	0.104	0.023	0.954
Limit of Detection (LOD, ∝g/L)			0.050	0.090	0.117	0.241	0.004	0.006	0.168	0.609	0.471	0.564	0.013	0.320	0.022	0.031	0.007	0.286
< LOQ replacement value†			0.109	0.194	0.253	0.522	0.009	0.013	0.365	1.320	1.020	1.222	0.028	0.693	0.047	0.067	0.015	0.620
< LOD replacement value‡			0.025	0.045	0.058	0.120	0.002	0.003	0.084	0.305	0.235	0.282	0.006	0.160	0.011	0.016	0.003	0.143

* Analysis failed and provided no data. † Replacement value determined as mean of LOQ and LOD. ‡ Replacement value determined as mean of LOD and zero.

molecules

MDPI

Table S9: Selected monoterpenes and C_{13}-norisoprenoids in control Chardonnay wine and Chardonnay wine with 0.4 g/L additions of marc extract from different varieties after 6-months of storage at 15 °C. Data expressed as a proportion of the total volatile compounds analysed in the analytical suite.

Sample	Category	Replicate	Proportion of total volatiles quantitated in analytical suite (%)															
			Limonene	1,8-Cineole	Terpinolene	Linalool	cis-Rose Oxide	trans-Rose Oxide	a-Terpineol	b-Citronellol	Nerol	Geraniol	cis-Vitispirane	TDN	b-Damascenone	a-Ionone	b-Ionone	Wine Lactone
Control Wine	Control	1	0.52	0.92	0.28	27.42	0.08	0.01	27.06	19.79	4.84	10.26	0.53	0.76	6.63	0.08	0.15	0.68
		2	0.80	0.92	0.28	28.77	0.04	0.01	26.75	19.74	4.84	9.49	0.58	0.76	6.13	0.08	0.14	0.68
		3*	-	-	-	-	-	-	-	-	-	-	-	-	-	-	-	-
2016 Gewurztraminer	Floral	1	0.10	0.19	0.06	39.87	0.16	0.02	30.70	2.79	5.05	12.37	1.06	0.15	7.24	0.06	0.02	0.14
		2	0.18	0.19	0.06	38.63	0.14	0.01	29.72	3.22	5.47	13.90	1.15	0.16	6.93	0.07	0.03	0.14
		3	0.20	0.19	0.06	41.24	0.12	0.01	29.51	2.79	4.15	12.50	1.47	0.16	7.36	0.07	0.04	0.14
Muscat Gordo	Floral	1	0.08	0.01	0.07	46.25	0.02	0.00	26.09	0.32	4.23	22.44	0.06	0.00	0.43	0.00	0.00	0.00
		2	0.09	0.01	0.09	49.48	0.02	0.00	25.35	0.35	3.91	20.20	0.07	0.00	0.44	0.00	0.01	0.00
		3	0.10	0.01	0.10	48.80	0.02	0.00	26.11	0.32	3.81	20.24	0.06	0.00	0.41	0.00	0.01	0.00
Muscat a PGB	Floral	1	0.12	0.02	0.14	52.54	0.08	0.01	32.64	0.32	4.15	9.10	0.11	0.02	0.74	0.01	0.00	0.01
		2	0.11	0.02	0.12	52.54	0.07	0.01	32.43	0.36	4.12	9.41	0.09	0.01	0.69	0.01	0.00	0.01
		3	0.07	0.02	0.08	54.33	0.07	0.01	30.62	0.38	3.97	9.65	0.09	0.02	0.67	0.00	0.00	0.01
Riesling	Floral	1	0.10	0.05	0.06	50.11	0.01	0.00	30.79	0.71	3.61	8.51	0.93	0.04	5.00	0.02	0.04	0.02
		2	0.09	0.06	0.08	51.19	0.01	0.00	32.47	0.90	2.41	6.64	1.05	0.05	4.94	0.02	0.04	0.05
		3*	-	-	-	-	-	-	-	-	-	-	-	-	-	-	-	-
Gewurztraminer	Floral	1	0.40	0.36	0.11	32.22	1.00	0.14	30.75	5.34	3.63	10.32	1.38	0.30	13.57	0.13	0.09	0.27
		2	0.21	0.37	0.11	32.15	0.92	0.13	30.64	5.98	3.48	10.05	1.51	0.30	13.65	0.13	0.09	0.27
		3	0.20	0.36	0.11	31.73	1.07	0.16	30.68	7.68	3.86	10.23	1.47	0.30	11.86	0.12	0.05	0.12
Viognier	Floral	1	0.09	0.05	0.06	52.30	0.03	0.01	31.44	1.17	3.42	8.85	0.23	0.04	2.24	0.02	0.03	0.02
		2	0.15	0.07	0.10	51.73	0.03	0.01	30.49	1.07	3.46	10.12	0.28	0.06	2.35	0.03	0.03	0.02
		3	0.14	0.07	0.10	51.54	0.04	0.00	31.29	1.64	3.64	9.00	0.30	0.06	2.06	0.03	0.04	0.05
Cabernet Sauvignon	Non-floral	1	0.30	0.54	0.70	17.09	0.06	0.01	22.36	7.42	2.84	7.00	2.57	0.45	38.03	0.19	0.04	0.40
		2	0.66	0.60	0.18	17.80	0.06	0.01	25.09	8.17	3.17	7.48	2.61	0.50	33.08	0.05	0.10	0.44
		3	0.35	0.62	0.19	19.68	0.07	0.01	26.49	8.75	3.26	7.09	2.93	0.51	29.25	0.21	0.14	0.46
Chardonnay	Non-floral	1	0.26	0.47	0.14	28.49	0.06	0.01	27.94	5.78	2.47	7.78	1.80	0.39	23.77	0.16	0.14	0.35
		2	0.28	0.49	0.15	29.17	0.07	0.01	28.32	3.34	2.58	8.14	1.85	0.40	24.51	0.17	0.17	0.36
		3	0.47	0.46	0.60	29.50	0.06	0.01	27.63	6.04	2.44	8.37	1.74	0.38	21.62	0.16	0.15	0.34
Sauvignon Blanc	Non-floral	1	0.26	0.47	0.14	23.06	0.10	0.03	26.47	5.33	2.48	7.69	2.96	0.39	29.92	0.16	0.20	0.35
		2	0.26	0.45	0.14	20.81	0.10	0.01	26.27	6.95	2.39	7.19	2.96	0.37	31.45	0.16	0.17	0.33
		3	0.26	0.47	0.14	22.15	0.12	0.03	25.86	6.38	2.45	7.29	3.06	0.38	30.79	0.16	0.11	0.34
Semillon	Non-floral	1	0.28	0.50	0.65	24.08	0.09	0.01	28.40	7.52	2.63	7.85	3.21	0.41	23.73	0.17	0.08	0.37
		2	0.31	0.56	0.17	24.64	0.07	0.01	28.92	6.75	2.92	8.54	3.03	0.46	22.95	0.19	0.08	0.41
		3	0.31	0.55	0.16	25.65	0.08	0.01	28.37	7.91	2.90	7.58	4.05	0.45	21.21	0.19	0.16	0.41
Shiraz	Non-floral	1	0.35	0.63	0.19	25.56	0.11	0.01	29.54	4.29	3.32	8.40	3.29	0.52	23.02	0.22	0.10	0.47
		2	0.37	0.65	0.19	26.47	0.10	0.04	29.52	4.43	3.43	6.95	3.44	0.54	23.03	0.23	0.13	0.48
		3	0.35	0.63	0.19	26.25	0.10	0.01	29.21	8.88	5.53	3.95	3.27	0.52	20.34	0.22	0.10	0.46
Verdelho	Non-floral	1	0.37	0.32	0.42	42.86	0.05	0.00	34.53	2.17	3.62	8.10	1.59	0.26	5.28	0.11	0.08	0.24
		2	0.31	0.30	0.39	42.61	0.04	0.00	33.01	3.88	4.34	7.99	1.53	0.25	4.93	0.10	0.08	0.22
		3	0.32	0.29	0.38	45.18	0.04	0.00	34.20	1.97	4.24	5.78	1.48	0.24	5.56	0.02	0.08	0.21

* Analysis failed and provided no data.

molecules

MDPI

Table S10: Concentration of selected monoterpenes and norisoprenoids in Chardonnay wine with 0.4 g/L additions of either floral or non-floral marc extracts, analysed after six-months of storage at 15 ºC. Data expressed as mean of all replicates ± standard deviation, with the ANOVA p-value and Tukey 95% honest significant difference (HSD) for each compound.

Extract type	Limonene	Linalool	cis-Rose oxide	a-Terpineol	b-Citronellol	Nerol	Geraniol	cis-Vitispirane	b-Ionone	b-Damascenone
Floral	0.91±1.15	465.74±638.77	0.38±0.28	262.73±340.7	4.89±3.4	37.46±53.18	163.08±287.8	1.48±0.91	0.09±0.06	9.81±4.47
Non-floral	0.14±0.04	11.38±7.41	0.03±0.01	11.3±4.82	2.45±0.84	1.29±0.6	2.95±0.94	0.93±0.34	0.05±0.02	7.91±3.83
p-value	0.0049	0.003	<0.0001	0.0022	0.0037	0.0044	0.0175	0.0174	0.0082	0.1738
Tukey 95% HSD	0.52	289.26	0.13	154.29	1.59	24.08	130.32	0.45	0.03	2.77

Compounds not shown - terpinolene, *trans*-rose oxide, 1,8-cineole only quantifiable in floral samples; TDN, a-ionone, wine lactone not quantifiable in any samples

Table S11: Mean concentration of most abundant monoterpenes in replicate wines resulting from addition of extracts. Data expressed as concentration as analysed in the wines; concentration above that present in the control wines; volatile concentration expressed as a weight-to-weight of the added extract.

Sample	Extract spike (g/L)	Mean concentration in in wine (∝g/L)*				Concentration in wine above control (∝g/L)				Concentration in wine (∝g/g of extract)			
		Geraniol	Linalool	Nerol	a-Terpineol	Geraniol	Linalool	Nerol	a-Terpineol	Geraniol	Linalool	Nerol	a-Terpineol
Control Wine	N/A	2.08	5.92	1.02	5.67	-	-	-	-	-	-	-	-
Muscat a PGB	0.396	99.1	560.68	43.06	336.62	97.02	554.76	42.04	330.95	244.95	1400.64	106.14	835.57
Viognier	0.405	28.21	158.29	10.65	94.91	26.13	152.37	9.63	89.24	64.52	376.25	23.78	220.36
Riesling	0.402	26.99	177.61	10.82	110.72	24.91	171.69	9.80	105.05	61.90	426.64	24.35	261.04
Muscat Gordo	0.402	760.18	1743.91	144.39	936.46	758.10	1737.99	143.37	930.79	1886.49	4324.88	356.77	2316.21
Gerwurztraminer	0.396	5.44	17.08	1.95	16.36	3.36	11.16	0.93	10.69	8.48	28.16	2.35	26.97
Chardonnay	0.400	3.31	11.88	1.02	11.43	1.23	5.96	0.00	5.76	3.08	14.90	0.00	14.40
Semillon	0.404	4.65	27.91	2.61	21.72	2.57	21.99	1.59	16.05	6.36	54.42	3.93	39.72
Verdelho	0.402	3.09	9.2	1.02	10.96	1.01	3.28	0.00	5.29	2.51	8.16	0.00	13.17
Sauvignon Blanc	0.399	1.96	7.95	1.25	8.97	-0.12	2.03	0.23	3.30	-0.30	5.08	0.58	8.26
Shiraz	0.403	2.9	8.99	1.02	10.37	0.82	3.07	0.00	4.70	2.04	7.63	0.00	11.68
Cabernet Sauvignon	0.400	2.38	6.01	1.02	8.13	0.30	0.09	0.00	2.46	0.75	0.22	0.00	6.15

* Mean of the replicates as shown in Table S8

Table S12: Mean concentration of bound and volatile monoterpenes from marc extracts, including sum of selected monoterpenes shown in this table and the correlation between the measures. Concentration data are repeated or aggregated from previous tables for clarity.

Sample	Concentration (∝g/g of extract)								
	In extract	From hydrolysis of extract			Evolving after storage of extract in wine for six months				
	Geraniol glucoside	Linalool	a-Terpineol	Sum#	Geraniol	Linalool	Nerol	a-Terpineol	Sum#
Muscat a PGB	1200.99	437.6	766.5	1204.0	244.95	1400.64	106.14	835.57	2587.31
Viognier	216.66	90.5	118.3	208.9	64.52	376.25	23.78	220.36	684.92
Riesling	185.66	213.8	256.6	470.5	61.90	426.64	24.35	261.04	773.93
Muscat Gordo	2082.21	1909.0	1677.2	3586.2	1886.49	4324.88	356.77	2316.21	8884.35
Gerwurztraminer	1305.81	161.2	153.3	314.5	8.48	28.16	2.35	26.97	65.95
Chardonnay	2.36*	13.1	20.1	33.3	3.08	14.90	0.00	14.40	32.38
Semillon	44.60	62.8	43.3	106.1	6.36	54.42	3.93	39.72	104.44
Verdelho	27.43	69.5	48.9	118.4	2.51	8.16	0.00	13.17	23.85
Sauvignon Blanc	85.00	111.9	78.8	190.6	0.00^	5.08	0.58	8.26	13.92
Shiraz	93.33	34.5	30.8	65.3	2.04	7.63	0.00	11.68	21.34
Cabernet Sauvignon	12.51*	35.0	26.7	61.7	0.75	0.22	0.00	6.15	7.12
		Pearson correlation coefficients (R)							
Extract	Geraniol glucoside	0.837	0.875	0.861	0.792	0.831	0.831	0.836	0.827
Hydrolysis	Linalool		0.974	0.994	0.994	0.989	0.992	0.984	0.992
	a-Terpineol			0.993	0.951	0.99	0.987	0.993	0.986
	Sum				0.98	0.996	0.996	0.995	0.996
In bottle	Geraniol					0.981	0.986	0.973	0.987
	Linalool						0.999	0.999	0.999
	Nerol							0.998	1.000
	a-Terpineol								0.998

* replaced by values representing the mean of the analytical LOQ and LOD, or LOD and zero

^ geraniol in wine with Sauvignon Blanc extract was lower than the unspiked control wine

sum of the monoterpenes shown in this table
